# Chemical Exposures: Not Immune to PFOS Effects?

**DOI:** 10.1289/ehp.116-a290a

**Published:** 2008-07

**Authors:** Kellyn S. Betts

Two recent studies have demonstrated that exposure to perfluorooctanyl sulfonate (PFOS) can alter mammalian immune system function. In one study, PFOS induced immunotoxicity in test animals at exposures analogous to those reported in the U.S. population, raising the possibility that “if humans and mice share similarities in their immune systems, then people today could be immunocompromised due to current PFOS exposure,” says Jennifer Keller, a research biologist at the National Institute of Standards and Technology’s Hollings Marine Laboratory. However, resolving this question will require carefully controlled epidemiologic studies that include relevant immune system and infectious disease end points.

PFOS was removed from 3M’s Scotchgard™ stain repellent by the end of 2002, but the compound degrades slowly and is dispersed widely throughout the environment. Data from the National Health and Nutrition Examination Survey 2003–2004 documented that it was found in 99.9% of samples.

Research slated for publication in the 1 July 2008 issue of *Toxicological Sciences* documents that production of the immunoglobulin M (IgM) antibody decreased in B6C3F_1_ mice exposed to PFOS. This suggests the immune response to pathogens may be suppressed and could result in increased disease susceptibility, says lead author Margie Peden-Adams, a research assistant professor at the Medical University of South Carolina. She says these effects occurred at serum PFOS levels (91.5 ppb in male mice) that were 14 times lower than the average blood concentrations of occupationally exposed humans and in the upper range of concentrations reported for the general population, making this the lowest reported lowest-observable-effect level for PFOS. She adds that suppression of IgM production in female mice occurred at dose concentrations 10 times higher than in males.

The male mice’s natural killer (NK) cell population, an important part of the innate immune system for tumor and viral surveillance, was increased, but female NK cell function was not affected. Peden-Adams speculates that the increase could be a compensation for a decrease in the male mice’s populations of cytotoxic T cells, which also have functions related to killing tumor cells. Additional testing determined that the cause of the decreased IgM production is probably an issue with B cells, the cell type that differentiates into antibody-producing cells, she says.

Another study, published 1 May 2008 in *Toxicological Sciences* by Deborah Keil, an associate professor of clinical laboratory sciences at the University of Nevada at Las Vegas, also linked a reduction in IgM antibody response to PFOS exposure. This study, which evaluated the immune systems of B6C3F_1_ mouse pups whose mothers were exposed to the compound during pregnancy, showed that NK cell function was decreased in male pups. Both studies document that PFOS targeted antibody production and that males appeared more sensitive than females to the effects of PFOS, Keil says.

Peden-Adams says her group was quite surprised by their findings and that further research is needed to determine the effects’ mode of action in order to determine human risk. Her paper posits that sex differences in sensitivity may be related to levels of estrogen and testosterone, because these steroids are known to affect immune system function. “We know that PFOS does have some endocrine-disrupting activity,” she says, adding that some research suggests the chemical may be antiestrogenic.

A third study using a different mouse strain found similar effects for perfluorooctanoic acid (PFOA). The study, published in the May 2008 issue of *EHP* by research biologist Robert Luebke of the U.S. Environmental Protection Agency National Health and Environmental Effects Research Laboratory, showed that adult female C57BL/6J mice exposed to higher concentrations of PFOA also exhibited a reduction in IgM production.

